# Supplementary material for: Effects of Ambient Temperature, Relative Humidity, and Precipitation on Diarrhea Incidence in Surabaya
Source: Int J Environ Res Public Health. 2023 Jan 28;20(3):2313. doi: 10.3390/ijerph20032313 (PMC9916310; doi:10.3390/ijerph20032313)
Supplement: Supplementary file 1 [file ijerph-20-02313-s001.zip › Supplementary figure.pdf]

Effects of Ambient Temperature, Relative Humidity and Precipitation on Diarrhea Incidence in Surabaya

Bima Sakti Satria Wibawa [1,†], Aussie Tahta Maharani [2,†], Gerry Andhikaputra [1], Marsha Savira Agatha Putri [3], Aditya Prana Iswara [1,4], Amir Sapkota [5], Ayushi Sharma [1,4], Arie Dipareza Syafei [6] and Yu-Chun Wang [1,7,*]

1 Department of Environmental Engineering, College of Engineering, Chung Yuan Christian University, 200 Chung-Pei Road, Zhongli, Taoyuan City 320314, Taiwan
2 Institute of Tropical Disease, Universitas Airlangga, Surabaya 60286, Indonesia
3 Department of Environmental Health, Faculty of Health Science, Universitas Islam Lamongan, Lamongan 62211, Indonesia
4 Department of Civil Engineering, College of Engineering, Chung Yuan Christian University, 200 Chung-Pei Road, Zhongli, Taoyuan City 320314, Taiwan
5 Department of Epidemiology and Biostatistics, University of Maryland School of Public Health, Maryland, MD 20742, USA
6 Department of Environmental Engineering, Institut Teknologi Sepuluh Nopember, Surabaya 60111, Indonesia
7 Research Center for Environmental Changes, Academia Sinica, 128 Academia Road, Section 2, Nankang, Taipei 11529, Taiwan
* Correspondence: ycwang@cycu.edu.tw;
Tel.: +886-3-265-4916; Fax: 886-3-265-4949

† These authors contributed equally to this work.

Supplementary Figure Legend

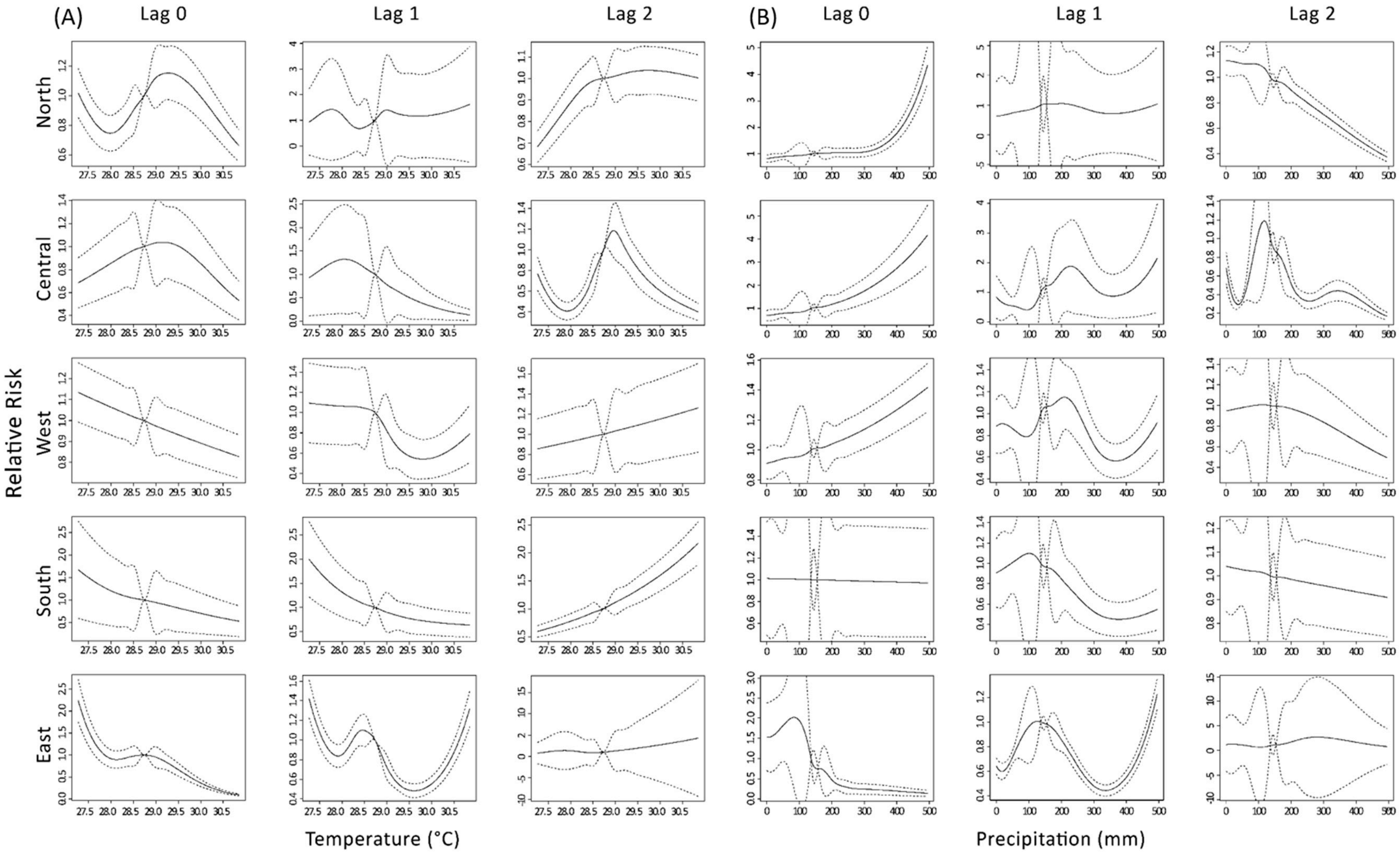

Supplementary Figure S1 Lag associations between the relative risk of diarrhea and monthly (A) average temperature (°C), and (B) cumulative precipitation (mm) in Surabaya from January 2018 to September 2020
