# Supplementary material for: Effects of Ambient Temperature, Relative Humidity, and Precipitation on Diarrhea Incidence in Surabaya
Source: Int J Environ Res Public Health. 2023 Jan 28;20(3):2313. doi: 10.3390/ijerph20032313 (PMC9916310; doi:10.3390/ijerph20032313)
Supplement: Supplementary file 1 [file ijerph-20-02313-s001.zip › Supplementary Tables.pdf]

Effects of Ambient Temperature, Relative Humidity and Precipitation on Diarrhea Incidence in Surabaya

Bima Sakti Satria Wibawa [1,†], Aussie Tahta Maharani [2,†], Gerry Andhikaputra [1], Marsha Savira Agatha Putri [3], Aditya Prana Iswara [1,4], Amir Sapkota [5], Ayushi Sharma [1,4], Arie Dipareza Syafei [6] and Yu-Chun Wang [1,7,*]

1 Department of Environmental Engineering, College of Engineering, Chung Yuan Christian University, 200 Chung-Pei Road, Zhongli, Taoyuan City 320314, Taiwan
2 Institute of Tropical Disease, Universitas Airlangga, Surabaya 60286, Indonesia
3 Department of Environmental Health, Faculty of Health Science, Universitas Islam Lamongan, Lamongan 62211, Indonesia
4 Department of Civil Engineering, College of Engineering, Chung Yuan Christian University, 200 Chung-Pei Road, Zhongli, Taoyuan City 320314, Taiwan
5 Department of Epidemiology and Biostatistics, University of Maryland School of Public Health, Maryland, MD 20742, USA
6 Department of Environmental Engineering, Institut Teknologi Sepuluh Nopember, Surabaya 60111, Indonesia
7 Research Center for Environmental Changes, Academia Sinica, 128 Academia Road, Section 2, Nankang, Taipei 11529, Taiwan
* Correspondence: ycwang@cycu.edu.tw;
Tel.: +886-3-265-4916; Fax: 886-3-265-4949

† These authors contributed equally to this work.

Supplementary Table Legend

Supplementary Table S1 Spearman correlation for each weather variables.

	Temperature	Precipitation	Relative humidity
Temperature	1	-	-
Precipitation	0.24	1	-
Relative humidity	-0.16*	0.87*	1

*p-value<0.05

Supplementary Table S2 Variance inflation factors in models.

	P-value	R^2	Temperature	Precipitation	Relative Humidity
Temperature + Precipitation + Relative Humidity	0.02	0.08	1.28	5.13	4.88
Temperature + Precipitation	0.05	0.02	1.03	1.01	-
Temperature + Relative Humidity	0.23	0.02	1.08	-	1.05

Supplementary Table S3 Region and lag-specific relative risk (95% confidence interval) of diarrhea associated with the average temperature and relative humidity at 5th and 95th percentiles relative to lowest risk measurement (28.7°C for temperature and 75.2% for relative humidity) from January 2018 to September 2020.

Lag	Regions of Surabaya				
	North	Central	West	South	East
Extreme low temperature (5th percentile)					
0	0.82 (0.59, 1.05)	1.21 (0.01, 2.39)	1.03 (0.83, 1.23)	1.63 (0.58, 2.67)	**3.22 (2.71, 3.74)**
1	0.45 (-0.67, 1.56)	0.94 (0.61, 1.28)	0.91 (0.75, 1.04)	1.03 (0.89, 1.16)	0.71 (0.61, 0.82)
2	0.57 (0.52, 0.62)	0.14 (0.11, 0.18)	0.95 (0.79, 1.11)	0.62 (0.51, 0.72)	2.09 (-0.76, 4.94)
Extreme high temperature (95th percentile)					
0	0.45 (0.33, 0.57)	0.45 (0.01, 0.91)	0.95 (0.77, 1.14)	0.56 (0.21, 0.92)	0.22 (0.18, 0.25)
1	6.24 (-9.28, 21.8)	0.09 (0.06, 0.12)	**1.32 (1.11, 1.53)**	**2.14 (1.86, 2.42)**	**5.39 (4.61, 6.17)**
2	0.79 (0.72, 0.86)	0.28 (0.21, 0.36)	1.09 (0.91, 1.27)	**2.06 (1.72, 2.41)**	1.61 (-0.58, 3.77)
Extreme low relative humidity (5th percentile)					
0	0.89 (0.69, 1.08)	1.84 (0.11, 3.58)	0.86 (0.72, 0.99)	0.93 (0.46, 1.41)	1.41 (0.65, 2.16)
1	0.22 (-0.79, 1.24)	0.71 (0.53, 0.86)	0.51 (0.38, 0.63)	0.34 (0.22, 0.46)	0.19 (0.06, 0.31)
2	**1.66 (1.51, 1.81)**	**1.14 (1.02, 1.27)**	0.96 (0.76, 1.15)	1.15 (0.96, 1.34)	1.19 (-1.36, 3.75)
Extreme high relative humidity (95th percentile)					
0	**2.08 (1.62, 2.53)**	7.85 (0.45, 15.3)	**2.13 (1.79, 2.47)**	1.06 (0.52, 1.59)	0.19 (0.09, 0.29)
1	1.61 (-5.75, 8.96)	**1.72 (1.31, 2.13)**	0.97 (0.73, 1.22)	0.71 (0.46, 0.95)	2.32 (0.81, 3.85)
2	0.52 (0.48, 0.57)	0.52 (0.46, 0.58)	0.37 (0.31, 0.45)	0.85 (0.71, 0.99)	0.37 (-0.42, 1.17)

Supplementary Table S4 Region and lag-specific relative risk (95% confidence interval) of diarrhea associated with the average temperature and precipitation at 5th and 95th percentiles relative to lowest risk measurement (28.7°C for temperature and 155 mm for precipitation) from January 2018 to September 2020.

Lag	Regions of Surabaya				
	North	Central	West	South	East
Extreme low temperature (5th percentile)					
0	1.02 (0.85, 1.18)	0.69 (0.47, 0.29)	1.13 (0.99, 1.28)	1.67 (0.59, 2.75)	**2.24 (1.75, 2.73)**
1	0.93 (-0.36, 2.22)	0.93 (0.11, 1.75)	1.09 (0.69, 1.49)	**1.99 (1.22, 2.77)**	**1.42 (1.22, 1.61)**
2	0.68 (0.61, 0.76)	0.77 (0.61, 0.93)	0.86 (0.56, 1.15)	0.59 (0.49, 0.70)	0.79 (-1.73, 3.33)
Extreme high temperature (95th percentile)					
0	0.66 (0.56, 0.77)	0.53 (0.36, 0.69)	0.83 (0.72, 0.93)	0.53 (0.19, 0.87)	0.09 (0.07, 0.11)
1	1.62 (-0.63, 3.87)	0.13 (0.02, 0.25)	0.79 (0.51, 1.07)	0.63 (0.39, 0.88)	**1.32 (1.14, 1.5)**
2	0.99 (0.9, 1.11)	0.39 (0.32, 0.48)	1.26 (0.82, 1.69)	**2.17 (1.79, 2.56)**	4.33 (-9.35, 18)
Extreme low precipitation (5th percentile)					
0	0.82 (0.69, 0.95)	0.69 (0.48, 0.91)	0.91 (0.81, 1.02)	1.01 (0.49, 1.53)	1.54 (0.71, 2.38)
1	0.63 (-0.53, 1.79)	0.83 (0.12, 1.54)	0.88 (0.64, 1.13)	0.91 (0.57, 1.24)	0.64 (0.57, 0.71)
2	**1.13 (1.02, 1.24)**	0.69 (0.52, 0.85)	0.95 (0.56, 1.33)	1.04 (0.85, 1.23)	1.21 (-4.22, 6.61)
Extreme high precipitation (95th percentile)					
0	**2.18 (1.83, 2.54)**	**3.05 (2.09, 4.01)**	**1.31 (1.16, 1.45)**	0.98 (0.48, 1.48)	0.18 (0.08, 0.28)
1	0.81 (-0.68, 2.27)	1.14 (0.17, 2.11)	0.64 (0.46, 0.82)	0.47 (0.29, 0.64)	0.65 (0.59, 0.72)
2	0.48 (0.43, 0.53)	0.31 (0.24, 0.39)	0.61 (0.36, 0.86)	0.93 (0.76, 1.11)	1.41 (-4.96, 7.76)
